# Enduring prenatal androgen effects on the female brain

**DOI:** 10.1093/braincomms/fcaf396

**Published:** 2025-10-14

**Authors:** Florian Kurth, Christian Gaser, Debra Spencer, Ajay Thankamony, Ieuan A Hughes, Helen Simpson, Umasuthan Srirangalingam, Helena Gleeson, Melissa Hines, Eileen Luders

**Affiliations:** School of Psychology, University of Auckland, Auckland 1142, New Zealand; Department of Diagnostic and Interventional Radiology, Jena University Hospital, Jena 07747, Germany; Department of Neurology, Jena University Hospital, Jena 07747, Germany; Department of Psychiatry and Psychotherapy, Jena University Hospital, Jena 07747, Germany; Department of Psychology, University of Cambridge, Cambridge CB2 1TN, UK; Department of Paediatrics, Addenbrooke's Hospital, University of Cambridge, Cambridge CB2 0QQ, UK; The Weston Centre for Paediatric Endocrinology and Diabetes, Addenbrooke's Hospital, University of Cambridge, Cambridge CB2 0QQ, UK; Department of Paediatrics, Addenbrooke's Hospital, University of Cambridge, Cambridge CB2 0QQ, UK; Department of Endocrinology and Diabetes, University College Hospital London, London NW1 2BU, UK; Department of Endocrinology and Diabetes, University College Hospital London, London NW1 2BU, UK; Queen Elizabeth Hospital, Birmingham B15 2GW, UK; Department of Psychology, University of Cambridge, Cambridge CB2 1TN, UK; School of Psychology, University of Auckland, Auckland 1142, New Zealand; Department of Women’s and Children’s Health, Uppsala University, Uppsala SE-751 05, Sweden; Swedish Collegium for Advanced Study (SCAS), Uppsala 75238, Sweden; Laboratory of Neuro Imaging, School of Medicine, University of Southern California, Los Angeles 90033, USA

**Keywords:** congenital adrenal hyperplasia, development, gender, machine learning, sex

## Abstract

Structural sex differences in the brain have been reported as early as infancy. Prenatal androgens have been hypothesized to contribute to these sex differences but the available evidence is inconclusive. Congenital adrenal hyperplasia (CAH) is a genetic variant that causes high levels of androgens during gestation in females and more male-typical behaviour after birth, whereas androgen levels and behaviour in males with CAH are largely typical. To assess whether prenatal androgens affect brain anatomy, we studied the largest CAH sample to date, matched pair-wise to a control group for sex, age, education and verbal intelligence. Anatomical sex differences were quantified using T1-weighted brain scans and a relevance-vector machine, which generated a continuous probabilistic brain sex index. Females with CAH exhibited significantly more male-like brain characteristics than control females, whereas no differences were observed between males with CAH and control males. This observed shift towards a male-typical brain anatomy in females with CAH supports the hypothesis that prenatal androgen exposure has formative effects on the human brain that persist into adulthood.

## Teaser

Using a continuous probabilistic brain sex index, Kurth *et al*. observed that women with congenital adrenal hyperplasia, but not men with congenital adrenal hyperplasia, exhibited more male-like brain characteristics than sex-matched controls. This suggests that prenatal androgens shape the human brain, with effects persisting into adulthood.

## Graphical Abstract

**Graphical Abstract:**
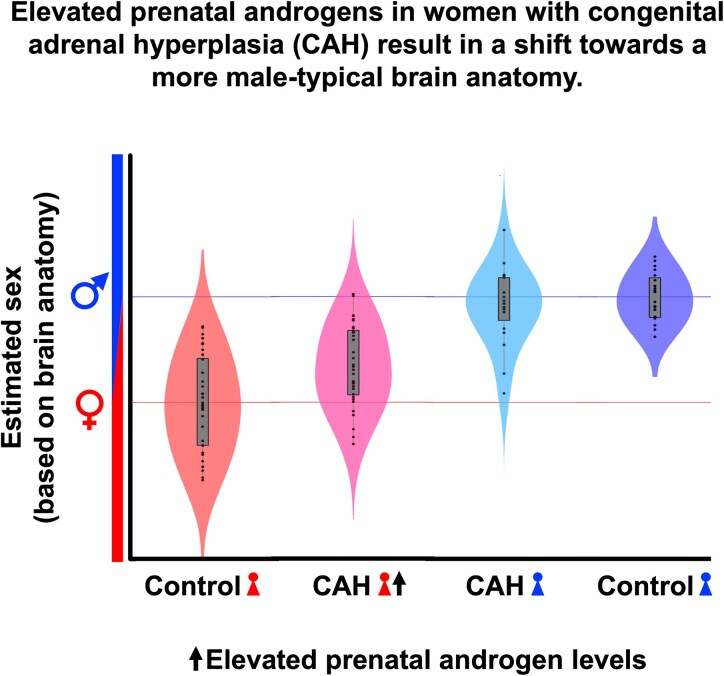

## Introduction

Studies of non-human mammals have demonstrated that sex hormones during early development contribute to sex-typical characteristics, with lasting effects into adulthood.^1-7^ More specifically, early testosterone exposure in female animals increases subsequent male-typical behaviour and decreases subsequent female-typical behaviour, with similar effects on brain structure.^1-6^ Some evidence for a similar link in humans, at least with respect to behaviour, comes from research on individuals with congenital adrenal hyperplasia (CAH). CAH is a genetic variant that results in high concentrations of androgens (including testosterone) during gestation in females, but largely normal concentrations of androgens in males.^3^ Females with CAH show increased male-typical preferences for toys, playmates and sexual partners as well as reduced satisfaction with a female gender identity, while no such effects are evident in males with CAH.^1-4^ In contrast to the outcomes of these behavioural studies, the few neuroimaging studies on CAH have not yet yielded evidence that prenatal androgen exposure results in more male-typical brains.^3,8^ However, definite conclusions are difficult because existing CAH research was based on small sample sizes^3^ and potentially under-powered studies (i.e. any significant effects might be small in size as also shown for a number of sex differences independent of CAH^9,10^).

Here we analysed data from the largest group of people with CAH who have been studied to date^8^ (*n* = 106), including 53 women and men with CAH who were matched to 53 control women and men. More specifically, we applied a novel multivariate machine learning approach capturing the overall pattern of sex-specific characteristics throughout the brain.^11,12^ We hypothesized that women with CAH have a more male-typical brain anatomy than control women but a more female-typical brain anatomy than control men. In contrast, given the lack of differences in behaviour between men with CAH and control men as well as the largely normal prenatal androgen levels in men with CAH,^3^ we expected a similar brain structure in men with CAH and in control men.

## Methods

### Study sample

A total of 53 individuals with classical CAH^13^ (33 females and 20 males), aged between 18 and 45 years (mean ± SD: 30.14.85 ± 7.90 years) and 53 controls (33 females and 20 males), aged between 18 and 45 years (mean ± SD: 30.35 ± 7.67 years) participated in the study. All participants were recruited in the UK, through National Health Service clinics, a national CAH support group, as well as flyers and advertisements posted in hospitals, general practice clinics or online. Participants with CAH were matched to controls with respect to sex, age, level of education and verbal intelligence, the last determined using the advanced vocabulary test^14^ (Table 1). All participants were required to be free from neurological or psychiatric disorders and to have no contraindications to MRI. All participants provided their informed consent. Approval for the study was obtained from the National Health Service Research Ethics Committee and the Health Research Authority in the UK (protocol number 15/EM/0532) and from the University of Auckland Human Participants Ethics Committee in New Zealand (protocol number 020825).

**Table 1:** Group demographics

	Control women	Women with CAH	Men with CAH	Control men
*N*	33	33	20	20
Age (years)	31.8 ± 8.5 (18.3–45.3)	31.1 ± 8.6 (18.3–45.7)	28.5 ± 6.6 (19.3–43.4)	27.9 ± 5.5 (19.4–40.8)
Verbal Intelligence**^a^**	6.3 ± 2.3 (1.8–11.0)	6.3 ± 2.6 (1.5–11.2)	5.6 ± 3.4 (2.0–12.5)	6.4 ± 3.1 (−1.0–13.5)
Highest level of education				
GCSEs	*n* = 6	*n* = 6	*n* = 4	*n* = 3
A levels	*n* = 6	*n* = 5	*n* = 7	*n* = 5
Vocational training	*n* = 5	*n* = 6	*n* = 1	*n* = 4
Bachelor’s degree	*n* = 12	*n* = 14	*n* = 5	*n* = 7
Master’s degree	*n* = 4	*n* = 2	*n* = 3	*n* = 1

GCSEs, General Certificates of Secondary Education.

^a^Measured using the advanced vocabulary test.

### Image acquisition, image processing and brain sex estimation

All brain images were acquired on a Siemens 3 Tesla Skyra system with a 32-channel head coil using the following parameters: TR = 2300 ms, TE = 2.98 ms, flip angle = 9°, matrix size = 256 × 240, 176 sagittal sections, voxel size = 1 × 1 × 1 mm^3^. These T1-weighted images underwent preprocessing via the CAT12 toolbox^15^ (version 12.8) and SPM12 (r7771). More specifically, the images were corrected for magnetic field inhomogeneities and tissue-classified into grey matter, white matter and cerebrospinal fluid, as previously detailed.^11,12^ In addition, TIV was calculated as the sum of the whole-brain tissue volumes of grey matter, white matter and cerebrospinal fluid. Subsequently, the grey and white matter partitions were spatially normalized to MNI space using 12-parameter affine transformations. Finally, the normalized grey and white matter segments were smoothed using an 8-mm FWHM Gaussian kernel, and the image resolution was set to 8 mm.

The resulting images were then used to classify each brain’s sex using a relevance vector regression machine,^16^ as detailed elsewhere.^11,12^ The classifier was trained on an independent training set of 528 participants (299 females and 229 males) obtained from the IXI Database (https://brain-development.org/ixi-dataset).^12^ The input for the sex classification in the current study are the voxel-wise tissue volumes, after performing a data reduction using PCA.^11,12^ The output of the sex classification is the so-called ‘brain sex index’, a number representing the degree of femaleness/maleness on a continuum, with 0 indicating the average female brain and 1 the average male brain.^11,12^

### Statistical analysis

All analyses were performed in MATLAB using a general linear model. First, differences in brain sex were assessed using a two-way ANCOVA, where the brain sex index was the dependent variable, group (CAH/control) and sex (male/female) the dependent variables and age as well as total intracranial volume the covariates. The significant main effects and interaction were then followed up by comparing each of the four groups (control women, women with CAH, men with CAH, control men) with each other in the same model, which resulted in six two-tailed *post hoc* tests. To safeguard against an inflated type 1 error, a Bonferroni correction was applied setting the threshold for significance at *P* < 0.0083 (i.e. 0.05/6) for the post hoc tests. The assumptions for parametric statistics were met by employing appropriate corrections for violations of non-sphericity^17^ and confirming the normal distribution of the residuals by a Lilliefors test.

Preceding the main analysis described above, the performance of the sex classifier was evaluated. For this purpose, the brain sex indices of the control sample were used to calculate the receiver-operator characteristic and its area under the curve. In addition, the brain sex index was binarized, with values larger than 0.5 labelled as 1 (male) and smaller than 0.5 as 0 (female). These binarized classifications were then used to calculate the classification accuracy (i.e. the number of correctly classified brains divided by all classified brains) when simply rounding the brain sex index.

## Results

**Figure 1:**
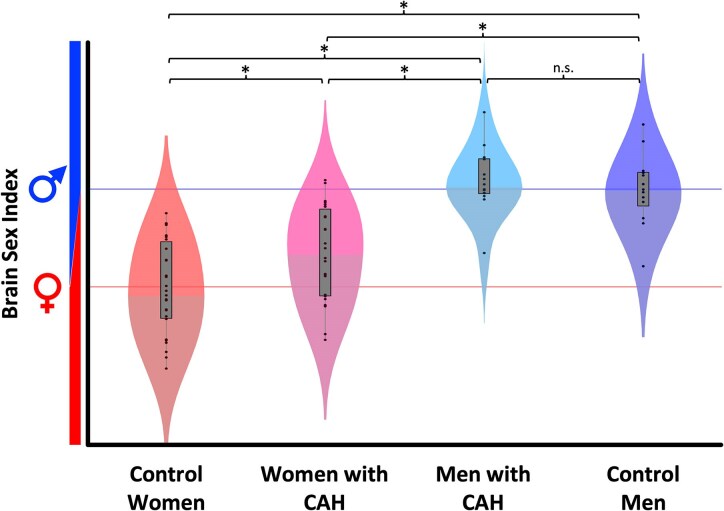
**Estimated sex based on brain anatomy in individuals with CAH and matched controls.** Violin plots depict the distribution of the brain sex indices for each group [control women (*n* = 33), women with CAH (*n* = 33), men with CAH (*n* = 20), control men (*n* = 20)]. The black dots show the individual indices, the gray boxes the group-specific interquartile range and the whiskers the group-specific 1.5 interquartile range; the difference in shading indicates the median. The red (blue) line is the average female (male) brain sex index in the control group. The average brain sex index of women with CAH ranged between control women and control men and was significantly different from both groups after Bonferroni correction (*P* ≤ 0.0083). In contrast, the difference between the two male groups (men with CAH versus control men) was not significant (n.s.). Statistical details are given in Table 2.

The performance of the sex classifier was evaluated using the control sample; the classifier achieved an accuracy of 88.7% and an area under the curve of the receiver-operator characteristic of 0.992. The main effects of biological sex (female versus male) and group (CAH versus control) were significant (*F*[1,100] = 54.2; *P* < 0.001 and *F*[1,100] = 3.9; *P* = 0.031). In addition, there was a significant group-by-sex interaction (*F*[1,100] = 7.8; *P* = 0.003), and appropriate *post hoc* comparisons revealed neuroanatomical effects in accordance with our prediction: Women with CAH ranged in the middle between control women and control men (see Fig. 1) and differed significantly from both groups (see Table 2). In contrast, men with CAH did not differ significantly from control men.

**Table 2:** Group differences with respect to the brain sex index (corrected for age and total intracranial volume)

Group comparisons	Effect size (Cohen’s *d*)	*t* (df)	Significance (*P*)
Control women versus control men	−1.75	−8.74 (100)	<0.001*
Control women versus women with CAH	−0.69	−3.47 (100)	0.003*
Women with CAH versus men with CAH	−0.82	−4.08 (100)	<0.001*
Control men versus men with CAH	0.13	0.65 (100)	0.414
Women with CAH versus control men	−0.94	−4.73 (100)	<0.001*
Control women versus men with CAH	−1.58	−7.80 (100)	<0.001*

^*^Significant after Bonferroni correction.

## Discussion

Animal studies have led to the postulation of a sensitive period during early development where androgens have an organizational impact on the human brain.^5-7,18^ Studies of individuals with CAH can provide evidence regarding the effect of natural variations in prenatal androgen exposure on human development^1,3^ because CAH involves atypically high concentrations of androgens during gestation in females but not in males.^3^ This is demonstrated in behavioural studies; for example, in comparison to same-sex controls, girls and women with CAH exhibit increased male-typical and reduced female-typical characteristics and behaviours, including toy and playmate preferences, sexual orientation and gender identity.^2,3^ In contrast, boys and men with and without CAH typically do not differ with respect to these characteristics. Thus, if androgens have an organizational impact on the human brain (i.e. as suggested by animal studies and by behavioural studies in humans), one would expect differences in brain anatomy when comparing women with CAH to control women (women with CAH would have more male-typical brains), but not when comparing men with CAH to control men.

Interestingly, this was never confirmed in humans in prior structural neuroimaging studies, where effects of CAH were either observed in both sexes or where women with CAH did not appear more male-typical.^3^ This may be explained by small sample sizes (CAH is a rare disorder occurring only in approximately 1 in 15 000 live births^8^) and/or by the magnitude of sex differences for specific brain regions (effect sizes are small in general^9,10^). Moreover, factors other than androgens, such as oestrogens, genes, glucocorticoids (which are used for treatment) as well as social factors,^1,3,13^ are likely to exert effects in addition. Consequently, any masculinizing effects due to prenatal androgens—that might be small to begin with—may remain below the threshold of detection. It is important to note though that while effect sizes on a univariate level might be small or even minute, a multitude of such small univariate effects might amount to large effects when regarded as a multivariate pattern (see Supplementary Fig. 1). Indeed, sex differences in *patterns* across the entire brain are larger and allow differentiating between male and female brains with high accuracy.^11,12,19-21^ In addition, when the expression of sex differences in these multivariate patterns is assessed as a continuous rather than binary variable, even partial effects as expected with prenatal androgen exposure can be detected. Another advantage of a continuous measure of sex differences is that it is in line with current theories that posit sexual differentiation to be the result of a complex interaction of genetic, hormonal, epigenetic and social factors.^5,22-26^

With respect to the outcomes of the current study, the observed lack of significant differences between men with CAH and control men is in line with reports of largely normal prenatal androgen levels in males with CAH.^3^ Conversely, the detected differences between women with CAH and control women is in line with reports of increased prenatal androgen levels in females with CAH.^1-4,13,27^ Furthermore, the current findings correspond to results from behavioural studies showing a more male-typical behaviour in girls and women with CAH but generally not in boys and men with CAH.^2,3^ While direct associations between a more male-typical brain anatomy and more male-typical behaviour remain to be established, our findings support the view that prenatal androgens play a role in shaping the human brain during early development. This may also explain (at least partly—as genetic and environmental influences will be at play as well^5,6^) why brains of boys and girls in the general population show structural differences even before the onset of puberty when sex hormone levels rise.^11,28,29^ Given that all participants in the present study were adults, the findings in women with CAH also suggest that the effects of prenatal androgen are enduring, which mirrors findings from animal studies testing the effects of early androgen exposure in non-human mammals.^1-7,18^ To our knowledge, this is the first study to provide neuroimaging evidence supporting theoretical predictions of masculinized brains in women with CAH and suggesting that prenatal androgens have lasting organizational effects on the human brain.

## Supplementary Material

fcaf396_Supplementary_Data

## Data Availability

The data that support the findings of this study are not publicly available due to ethics/IRB restrictions. Any reasonable request for data access should be made to the corresponding author. All analyses were performed in MATLAB 2022b (www.mathworks.com/products/matlab) using publicly available code, specifically the SPM12 toolbox (r7771; https://www.fil.ion.ucl.ac.uk/spm/), the CAT12 toolbox^15^ (version 12.8; https://neuro-jena.github.io/cat/), the Matlab Toolbox for Dimensionality Reduction (https://lvdmaaten.github.io/drtoolbox) and ‘The Spider’ (https://people.kyb.tuebingen.mpg.de/spider/main.html).

## References

[1] Hines M . Sex-related variation in human behavior and the brain. Trends Cogn Sci. 2010;14(10):448–456. 20724210 10.1016/j.tics.2010.07.005 PMC2951011

[2] Hines M . Neuroscience and sex/gender: Looking back and forward. J Neurosci. 2020;40(1):37–43. 31488609 10.1523/JNEUROSCI.0750-19.2019 PMC6939487

[3] Beltz AM, Demidenko MI, Wilson SJ, Berenbaum SA. Prenatal androgen influences on the brain: A review, critique, and illustration of research on congenital adrenal hyperplasia. J Neurosci Res. 2021;101(5):563–574. 34139025 10.1002/jnr.24900 PMC12124213

[4] Berenbaum SA, Beltz AM. How early hormones shape gender development. Curr Opin Behav Sci. 2016;7:53–60. 26688827 10.1016/j.cobeha.2015.11.011 PMC4681519

[5] McCarthy MM, Arnold AP. Reframing sexual differentiation of the brain. Nat Neurosci. 2011;14(6):677–683. 21613996 10.1038/nn.2834 PMC3165173

[6] Arnold AP . The organizational-activational hypothesis as the foundation for a unified theory of sexual differentiation of all mammalian tissues. Horm Behav. 2009;55(5):570–578. 19446073 10.1016/j.yhbeh.2009.03.011 PMC3671905

[7] Phoenix CH, Goy RW, Gerall AA, Young WC. Organizing action of prenatally administered testosterone propionate on the tissues mediating mating behavior in the female Guinea pig. Endocrinology. 1959;65:369–382. 14432658 10.1210/endo-65-3-369

[8] Khalifeh N, Omary A, Cotter DL, Kim MS, Geffner ME, Herting MM. Congenital adrenal hyperplasia and brain health: A systematic review of structural, functional, and diffusion magnetic resonance imaging (MRI) investigations. J Child Neurol. 2022;37(8-9):758–783. 35746874 10.1177/08830738221100886 PMC9464669

[9] Ritchie SJ, Cox SR, Shen X, et al Sex differences in the adult human brain: Evidence from 5216 UK biobank participants. Cereb Cortex. 2018;28(8):2959–2975. 29771288 10.1093/cercor/bhy109 PMC6041980

[10] Lotze M, Domin M, Gerlach FH, et al Novel findings from 2,838 adult brains on sex differences in gray matter brain volume. Sci Rep. 2019;9(1):1671. 30737437 10.1038/s41598-018-38239-2 PMC6368548

[11] Kurth F, Gaser C, Luders E. Development of sex differences in the human brain. Cogn Neurosci. 2021;12(3-4):155–162. 32902364 10.1080/17588928.2020.1800617 PMC8510853

[12] Kurth F, Gaser C, Sanchez FJ, Luders E. Brain sex in transgender women is shifted towards gender identity. J Clin Med. 2022;11(6):1582. 35329908 10.3390/jcm11061582 PMC8955456

[13] Merke DP, Auchus RJ. Congenital adrenal hyperplasia due to 21-hydroxylase deficiency. N Engl J Med. 2020;383(13):1248–1261. 32966723 10.1056/NEJMra1909786

[14] Ekstrom RB, French JW, Harman HH, Dermen D. Manual for kit of factor referenced cognitive tests. Educational Testing Service; 1976.

[15] Gaser C, Dahnke R, Thompson PM, Kurth F, Luders E, The Alzheimer's Disease Neuroimaging Initiative. CAT: a computational anatomy toolbox for the analysis of structural MRI data. Gigascience. 2024;13:giae049. 39102518 10.1093/gigascience/giae049 PMC11299546

[16] Tipping ME . Sparse Bayesian learning and the relevance vector machine. J Mach Learn Res. 2001;1:211–244.

[17] Glaser D, Friston K. Covariance components. In: Friston K, Ashburner J, Kiebel S, Nichols TE, Penny WD, eds. Statistical parametric mapping: The analysis of functional brain images. Elsevier; 2007:140–147. chap 10.

[18] MacLusky NJ, Naftolin F. Sexual differentiation of the central nervous system. Science. 1981;211(4488):1294–1302. 6163211 10.1126/science.6163211

[19] Rosenblatt JD . Multivariate revisit to “sex beyond the genitalia”. Proc Natl Acad Sci U S A. 2016;113(14):E1966–E1967. 26984492 10.1073/pnas.1523961113 PMC4833231

[20] Chekroud AM, Ward EJ, Rosenberg MD, Holmes AJ. Patterns in the human brain mosaic discriminate males from females. Proc Natl Acad Sci U S A. 2016;113(14):E1968. 26984491 10.1073/pnas.1523888113 PMC4833246

[21] Ryali S, Zhang Y, de Los Angeles C, Supekar K, Menon V. Deep learning models reveal replicable, generalizable, and behaviorally relevant sex differences in human functional brain organization. Proc Natl Acad Sci U S A. 2024;121(9):e2310012121. 38377194 10.1073/pnas.2310012121 PMC10907309

[22] Arnold AP, Burgoyne PS. Are XX and XY brain cells intrinsically different? Trends Endocrinol Metab. 2004;15(1):6–11. 14693420 10.1016/j.tem.2003.11.001

[23] Arnold AP, Chen X. What does the “four core genotypes” mouse model tell us about sex differences in the brain and other tissues? Front Neuroendocrinol. 2009;30(1):1–9. 19028515 10.1016/j.yfrne.2008.11.001 PMC3282561

[24] Carruth LL, Reisert I, Arnold AP. Sex chromosome genes directly affect brain sexual differentiation. Nat Neurosci. 2002;5(10):933–934. 12244322 10.1038/nn922

[25] De Vries GJ, Rissman EF, Simerly RB, et al A model system for study of sex chromosome effects on sexually dimorphic neural and behavioral traits. J Neurosci. 2002;22(20):9005–9014. 12388607 10.1523/JNEUROSCI.22-20-09005.2002 PMC6757680

[26] Arnold AP . Sexual differentiation of brain and other tissues: Five questions for the next 50 years. Horm Behav. 2020;120:104691. 31991182 10.1016/j.yhbeh.2020.104691 PMC7440839

[27] Merke DP, Fields JD, Keil MF, Vaituzis AC, Chrousos GP, Giedd JN. Children with classic congenital adrenal hyperplasia have decreased amygdala volume: Potential prenatal and postnatal hormonal effects. J Clin Endocrinol Metab. 2003;88(4):1760–1765. 12679470 10.1210/jc.2002-021730

[28] Gilmore JH, Lin W, Prastawa MW, et al Regional gray matter growth, sexual dimorphism, and cerebral asymmetry in the neonatal brain. J Neurosci. 2007;27(6):1255–1260. 17287499 10.1523/JNEUROSCI.3339-06.2007 PMC2886661

[29] Benavides A, Metzger A, Tereshchenko A, et al Sex-specific alterations in preterm brain. Pediatr Res. 2019;85(1):55–62. 30279607 10.1038/s41390-018-0187-5 PMC6353678

