# Supplementary material for: Enduring prenatal androgen effects on the female brain
Source: Brain Commun. 2025 Oct 14;7(6):fcaf396. doi: 10.1093/braincomms/fcaf396 (PMC12581814; doi:10.1093/braincomms/fcaf396)
Supplement: fcaf396_Supplementary_Data [file fcaf396_supplementary_data.pdf]

Supplementary Figure

Kurth et al., Enduring prenatal androgen effects on the female brain

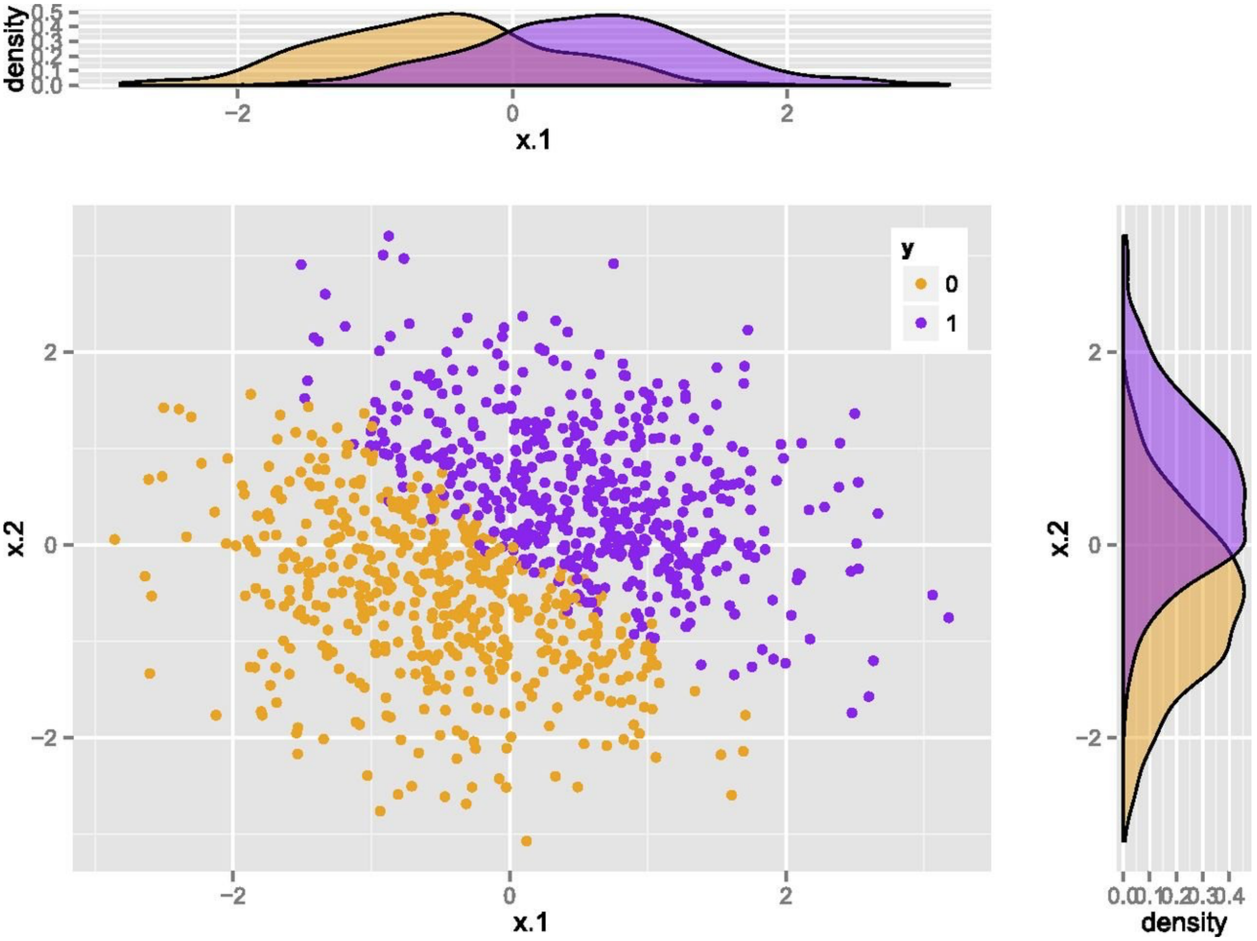

Supplementary Figure 1. While group differences may only be small (or even tiny) when using univariate measurements, these differences may become very large and groups may be perfectly separable when regarded as a multivariate pattern (e.g., using a classifier). Reprinted from Rosenblatt (2016)[1] with permission.

Reference

1. Rosenblatt JD. Multivariate revisit to "sex beyond the genitalia". *Proc Natl Acad Sci U S A* **113**, E1966-1967 (2016).
